# Dendritic Cell-Mediated Vaccination Relies on Interleukin-4 Receptor Signaling to Avoid Tissue Damage after *Leishmania major* Infection of BALB/c Mice

**DOI:** 10.1371/journal.pntd.0001721

**Published:** 2012-07-03

**Authors:** Anita Masic, Ramona Hurdayal, Natalie E. Nieuwenhuizen, Frank Brombacher, Heidrun Moll

**Affiliations:** 1 Institute for Molecular Infection Biology, University of Würzburg, Würzburg, Germany; 2 Institute of Infectious Disease and Molecular Medicine, University of Cape Town, Cape Town, South Africa; Institut Pasteur, France

## Abstract

Prevention of tissue damages at the site of *Leishmania major* inoculation can be achieved if the BALB/c mice are systemically given *L. major* antigen (LmAg)-loaded bone marrow-derived dendritic cells (DC) that had been exposed to CpG-containing oligodeoxynucleotides (CpG ODN). As previous studies allowed establishing that interleukin-4 (IL-4) is involved in the redirection of the immune response towards a type 1 profile, we were interested in further exploring the role of IL-4. Thus, wild-type (wt) BALB/c mice or DC-specific IL-4 receptor alpha (IL-4Rα)-deficient (CD11c^cre^IL-4Rα^−/lox^) BALB/c mice were given either wt or IL-4Rα-deficient LmAg-loaded bone marrow-derived DC exposed or not to CpG ODN prior to inoculation of 2×10^5^ stationary-phase *L. major* promastigotes into the BALB/c footpad. The results provide evidence that IL4/IL-4Rα-mediated signaling in the vaccinating DC is required to prevent tissue damage at the site of *L. major* inoculation, as properly conditioned wt DC but not IL-4Rα-deficient DC were able to confer resistance. Furthermore, uncontrolled *L. major* population size expansion was observed in the footpad and the footpad draining lymph nodes of CD11c^cre^IL-4Rα^−/lox^ mice immunized with CpG ODN-exposed LmAg-loaded IL-4Rα-deficient DC, indicating the influence of IL-4Rα-mediated signaling in host DC to control parasite replication. In addition, no footpad damage occurred in BALB/c mice that were systemically immunized with LmAg-loaded wt DC doubly exposed to CpG ODN and recombinant IL-4. We discuss these findings and suggest that the IL4/IL4Rα signaling pathway could be a key pathway to trigger when designing vaccines aimed to prevent damaging processes in tissues hosting intracellular microorganisms.

## Author Summary

Cutaneous leishmaniasis is endemic in tropical and subtropical regions of the world. Effective vaccination strategies are urgently needed because of the emergence of drug-resistant parasites and severe side effects of chemotherapy. We previously established a DC-based vaccination strategy to induce complete and long-lasting immunity to experimental leishmaniasis using *Leishmania major* antigen-loaded and CpG oligodeoxynucleotide-activated DC as a vaccine carrier. In the present study we investigated the role of IL-4Rα-mediated instruction of the vaccinating DC and the host DC during induction of protection against leishmaniasis. The results demonstrate that IL-4Rα signaling in DC used as vaccine carrier plays an important role in induction of protective immunity against *L. major* infection, as only mice vaccinated with IL-4 responder DC are able to trigger effective Th1-mediated immunity. The immunity is hallmarked by high levels of *L. major*-induced bioactive IL-12 production in the lymph nodes of vaccinated mice. Together, these findings suggest that IL-4 is a strong adjuvant to induce Th1-biased immunity against leishmaniasis and possibly other infections with intracellular pathogens, indicating that IL-4 needs to be considered in the development of efficient cell-mediated vaccination strategies.

## Introduction

*Leishmania spp.* infection in experimental mouse models provided insights into the polarization of immune responses against intracellular parasites, resulting either in self-healing local inflammation (e.g., C57BL/6) or severe and fatal leishmaniasis (e.g., BALB/c) [1], [2]. BALB/c mice fail to develop a protective interferon (IFN)-γ-mediated T helper (Th) 1 response to *Leishmania* infection [3], [4], but show a disease-promoting IL-4-driven Th2 response [5], [6].

Dendritic cells (DC) are migratory antigen-presenting cells (APC) which are highly specialized in uptake, processing and presentation of pathogen-derived antigens via major histocompatibility complex (MHC) molecules to T cells [7], resulting in the cytokine-regulated differentiation into Th1 or Th2 cells. Activated DC are characterized by high levels of MHC class II, CD80 and CD86 molecules [8], [9]. The ability of DC to release IL-12 in response to microbial stimuli is considered to be pivotal for the induction of Th1 responses [10], [11].

We previously demonstrated that the Toll-like receptor (TLR) 9 ligand CpG oligodeoxynucleotides (ODN) is a potent inducer of DC-derived IL-12, thus enabling DC to mediate complete and long-lasting immunity to experimental leishmaniasis. Prophylactic immunization with CpG ODN-activated and *Leishmania major* antigen (LmAg)-loaded BMDC one week or even 16 weeks prior to challenge has been shown to confer protection against *L. major* and, furthermore, these cured mice resist a secondary challenge 10 weeks after primary infection showing no sign of disease up to 20 weeks after rechallenge [12]. Interestingly, protection was not dependent on IL-12 secretion by the immunizing DC, as BALB/c mice treated with LmAg-loaded IL-12p35^−/−^ or IL-12p40^−/−^ DC were resistant against *L. major* infection, but the availability of recipient IL-12 was essential for the initiation of a protective immune response by DC, as neutralization of IL-12 during T cell priming diminished the protective effect of the vaccine [12].

The elaboration of DC-mediated vaccination strategies in animal models can be used as a tool to enhance the knowledge of the complex parasite-host interactions resulting in immunity against intracellular pathogens. It is well established that the main inducer of a Th2 response *in Leishmania*-susceptible BALB/c mice is IL-4 [13]. On the other hand, it has been shown that IL-4 has the ability to instruct a Th1 response and resistance against *L. major* in BALB/c mice. The presence of IL-4 during the initial phase of DC activation results in an increased IL-12-driven Th1 response [14].

To investigate the functional role of IL-4-mediated signaling during *Leishmania* infection, various knock-out mice have been generated. IL-4-deficient (IL-4^−/−^) [15], as well as IL-4 receptor alpha (IL-4Rα)-deficient BALB/c mice [16] are resistant to infection with *L. major*. Cell-specific IL-4Rα-deficient (IL-4Rα^−/−^) mice have been generated to investigate the impact of IL-4Rα-mediated signaling on various cell types during *Leishmania* infection. CD4^+^ T cell-specific Lck^cre^IL-4Rα^−/lox^ BALB/c mice show a resistant phenotype [17], whereas DC-specific CD11c^cre^IL-4Rα^−/lox^ BALB/c mice are hyper-susceptible (Hurdayal et al., manuscript in preparation). Hyper-susceptibility in CD11c^cre^IL-4Rα^−/lox^ mice is characterized by increased footpad swelling, the development of severe necrotic lesions and high parasite dissemination into organs, demonstrating that IL-4Rα signaling in DC is a necessity to control severe *Leishmania* infection.

In the present study, IL-4Rα^−/−^ bone marrow-derived DC (BMDC) from IL-4Rα^−/−^ BALB/c mice were used to investigate the effect of IL-4Rα-mediated signaling in DC used as vaccine carrier in *L. major*-susceptible mice. The results demonstrate that IL-4Rα signaling in BMDC plays an important role in the vaccine-mediated induction of protective immunity against *L. major* infection.

## Methods

### Ethical statement

All mice were kept under specific pathogen-free conditions. Mice experiments were performed in strict accordance with the German Animal Welfare Act 2006 (TierSchG) and the animal protocol was approved by the government of Lower Franconia (permission no. 55.2-2531.01-16/09) and by the Animal Research Ethics Committee of the University of Cape Town (license no. 009/042).

### Mice and parasites

Sex- and age-matched wild-type (wt) BALB/c (Charles River Breeding Laboratories, Sulzfeld, Germany) and CD11c^cre^IL-4Rα^−/lox^ BALB/c mice (Hurdayal et al., manuscript in preparation) were 6–8 weeks old at the onset of the experiments. IL-4Rα^−/−^ BALB/c [18] mice were kindly provided by Gottfried Alber (University of Leipzig, Germany).

The virulent *L. major* isolate (MHOM/IL/81/FE/BNI) was maintained by continuous passage in BALB/c mice. Amastigotes were isolated from lesions as previously described [19]. Promastigotes were grown *in vitro* in blood-agar cultures. For the preparation of LmAg, stationary-phase promastigotes were subjected to three cycles of rapid freezing and thawing and diluted to a final concentration of 1×10^9^ ml^−1^ in phosphate-buffered saline (PBS).

### Preparation of bone marrow-derived dendritic cells (BMDC)

DC were generated from bone marrow progenitors as described previously [20]. Briefly, isolated bone marrow cells from 6–8 week-old female BALB/c or IL-4Rα^−/−^ mice were cultured in RPMI 1640 medium (Invitrogen, Karlsruhe, Germany) in the presence of 200 U ml^−1^ recombinant mouse granulocyte-macrophage colony-stimulating factor (GM-CSF; PeproTech, London, United Kingdom). Fresh medium supplemented with GM-CSF was added to the culture on days 3 and 6. After 10 days, non-adherent cells were harvested and used for further experiments. These cells were shown to have the typical myeloid DC morphology [12]. BMDC were incubated for 4 hours in the presence of either 25 µg ml^−1^ CpG ODN 1668 (5′-TCCATGACGTTCCTGATGCT-3′, Qiagen Operon, Cologne, Germany) or 20 ng ml^−1^ recombinant mouse IL-4 (rIL-4; BD Biosciences, Heidelberg, Germany), or a combination of both, prior to the addition of LmAg for 18 hours. Thereafter, the BMDC were washed and resuspended at 5×10^6^ ml^−1^ in PBS.

### Treatment of mice

BALB/c and CD11c^cre^IL-4Rα^−/lox^ mice were treated with 5×10^5^ BMDC intravenously (i.v.) into the tail vein. Control mice were treated with PBS. One week post vaccination the mice were infected subcutaneously into the right hind footpad with 2×10^5^ stationary-phase *L. major* promastigotes in a final volume of 30 µl in PBS. The course of infection was monitored weekly by measuring the increase in footpad size of the infected versus the noninfected footpad.

One. 3 or 6 weeks post infection, mice were sacrificed and single cell suspensions from the infected footpads as well as the draining popliteal lymph nodes were obtained. The parasite burden was determined by limiting dilution assays as described previously [21].

### Flow cytometry

#### Extracellular FACS

Lymphocytes were fixed with paraformaldehyde (PFA, 4%) and resuspended in FACS buffer containing anti-Fc receptor antibodies (Ab) (purified rat anti-mouse CD16/CD32) together with the appropriate combinations of the following Ab: Biotin-streptavidin-horseradish peroxidase (SAV-HRP)-conjugated anti-CD11c (HL3); fluorescein isothiocyanate (FITC)-conjugated anti-I-A^d^ (AMS-32.1); and phycoerythrin (PE)-conjugated anti-CD80 (16-10A1) (all Ab were purchased from BD Biosciences, Heidelberg, Germany). Data was obtained using the FACSCalibur flow cytometer (BD Biosciences, Heidelberg, Germany) and analyzed using FlowJo (Tree Star Inc., CA, USA).

#### Intracellular FACS

1×10^6^ lymphocytes were activated for 2 hours with 25 ng ml^−1^ phorbol myristate acetate (PMA) and 1 µg ml^−1^ ionomycin (both from Sigma-Aldrich, Deisenhofen, Germany). Cultures were supplemented with 1 µM monensin for the final 4 hours of culture. Cells were stained with biotin-SAV-HRP-conjugated anti-CD11c (HL3) or FITC-conjugated anti-CD4 Ab and fixed in 4% PFA, permeabilized with 0.2% saponin and stained using PE-conjugated anti-IL-12 (C15.6), anti-IFN-γ XMG1.2) or anti-IL-4 Ab. IgG1 was used as isotope control (all Ab from BD Biosciences, Heidelberg, Germany).

### LmAg-stimulated cytokine release

5×10^6^ lymphocytes were cultured in the presence of LmAg (parasite-to-cell ratio 30∶1) or left untreated for 72 hours. The levels of IL-4, IL-12p70 and IFN-γ in the culture supernatants were determined by sandwich ELISA using Ab pairs purchased from BD Biosciences according to the manufacturer's instructions.

### Statistical analysis

Values are given as mean ± SD and significant differences were determined using Student's *t* test (GraphPad Prism version 5, San Diego, CA, USA).

## Results

### IL-4Rα signaling in BMDC used as vaccine carrier plays an important role in the induction of resistance against *L. major* infection

It has been shown that the Th2 key cytokine IL-4 can induce protective Th1-mediated immunity in *L. major*-susceptible BALB/c mice, as characterized by the secretion of high levels of DC-derived IL-12 [14]. In order to investigate whether IL-4Rα signaling in DC used as vaccine carrier is required to induce protection against leishmaniasis, BMDC were generated from IL-4Rα-deficient BALB/c mice or wt BALB/c mice. The BMDC were activated with the TLR 9 ligand CpG ODN and pulsed with LmAg prior to i.v. injection into naive BALB/c mice. Immunized BALB/c or control mice were challenged with *L. major* one week after vaccination, and the course of disease was monitored weekly.

**Figure 1:**
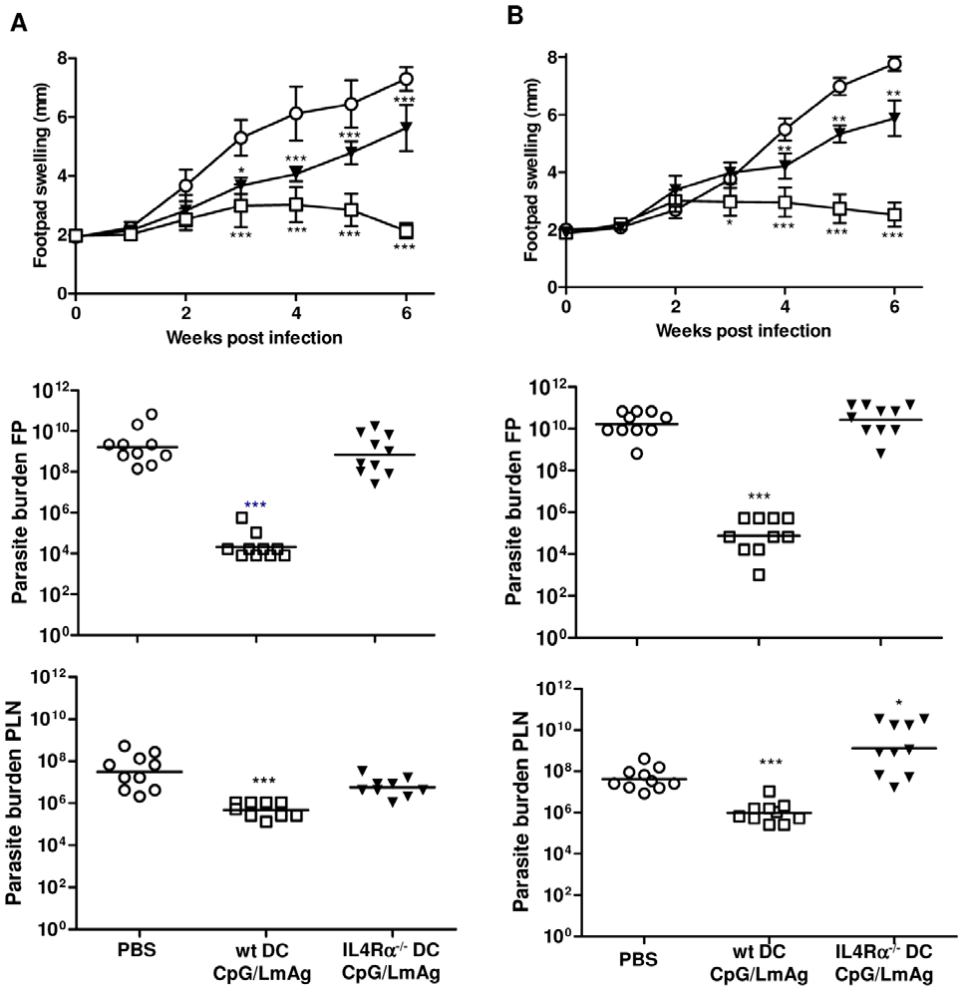
CpG ODN-activated and LmAg-loaded IL-4Rα^−/−^ BMDC fail to induce protection against leishmaniasis. BALB/c mice (**A**) or CD11c^cre^IL-4Rα^−/lox^ mice (**B**) were immunized i.v. with *in vitro* CpG ODN-activated and LmAg-loaded wt (□) or IL-4Rα^−/−^ (▾) BMDC one week prior to infection with *L. major* promastigotes. Control mice received PBS (○) before infection. The increase in size of the infected compared with the noninfected footpad was measured weekly. The results are expressed as mean ± SD of 10 animals. *, p<0.05, ***, p<0.0005 compared to PBS group (○). The parasite burden of the infected footpad and the draining popliteal lymph nodes of BALB/c and CD11c^cre^IL-4Rα^−/lox^ mice was determined six weeks after infection. The results of two independent experiments (10 mice) are shown. *, p<0.05, ***, p<0.0005 compared to the respective PBS-treated control group. PLN, popliteal lymph node. wt DC/CpG/LmAg, wild-type DC activated with CpG ODN and pulsed with *L. major*-antigen. IL-4Rα^−/−^ DC, interleukin-4 receptor alpha-chain-deficient dendritic cells.

In accordance with our previous study [12], mice immunized with CpG ODN-activated and LmAg-pulsed wt BMDC were able to control leishmaniasis. However, a significant progression of *L. major* infection was observed in mice immunized with CpG ODN-activated and LmAg-pulsed BMDC generated from IL-4Rα-deficient donors (Figure 1A). Even though these mice were able to restrict footpad swelling during the first three weeks, an uncontrolled lesion development was observed in the advanced phase of infection. Unprotected control mice showed a progressive course of disease with massive footpad swelling and development of necrotic lesions, which was not observed in mice immunized with conditioned IL-4Rα-deficient BMDC. The lack of necrotic lesions in these mice can most likely be explained by the delayed course of disease.

To determine whether the clinical outcome corresponds with the control of parasite replication, we analyzed the parasite burden in the infected footpads and the draining popliteal lymph nodes. The results revealed a significant correlation between parasite numbers and clinical outcome. The parasite burden at the site of infection was reduced about 10^5^-fold and within the draining lymph node about 10^2^-fold in protected mice (wt DC/CpG/LmAg) compared to unprotected mice. BALB/c mice immunized with IL-4Rα-deficient BMDC developed severe and progressive leishmaniasis, even though the footpad swelling did not reach the levels of PBS-treated mice. The parasite burden at the lesion site was not affected, but a 10-fold reduction of parasite burden was observed in the infected PLN. Equivalent results were obtained 3 weeks post infection (data not shown). Together, these findings demonstrate that IL-4Rα-deficient BMDC were unable to induce parasite clearance in the host organism.

The above results showed the importance of IL-4Rα-mediated instruction of DC used as vaccine carrier. To investigate whether DC of the host also require IL-4Rα signaling during vaccination, we immunized and infected DC-specific IL-4Rα^−/−^ BALB/c mice (CD11c^cre^IL-4Rα^−/lox^) (Figure 1B). Wt DC loaded only with LmAg or CpG had no protective effect (data not shown), as expected. The treatment of mice with conditioned wt BMDC induced protection independent of whether DC of the host organism are IL-4 responders (Figure 1A) or not (Figure 1B). In contrast, immunization with conditioned IL-4Rα^−/−^ BMDC was not capable to induce the control of infection, as indicated by uncontrolled lesion development and parasite burden at the site of infection. We observed controlled footpad swelling in mice treated with PBS or BMDC until 3 weeks post infection (Figure 1B). Delayed lesion development was accompanied by reduced parasite burden and high IFN-γ response by LmAg-stimulated draining LN cells 3 weeks post infection, but a Th1-biased immunity was not established during the onset of infection (data not shown). In a complete IL-4Rα-deficient system (neither vaccine carrier nor recipient DC are IL-4 responders), an uncontrolled parasite replication in the infected lymph nodes (10^2^-fold increase compared to PBS group) was observed. This observation is in contrast to the 10-fold reduction of parasite burden within wt BALB/c mice immunized with IL-4Rα-deficient BMDC (Figure 1A), indicating that the inhibition of IL-4Rα signaling on host DC is detrimental and leads to increased dissemination of parasites into lymph nodes. These results indicate the importance of IL-4Rα-mediated instruction of DC used as vaccine carrier to mediate protection against leishmaniasis.

### Activation of LmAg-pulsed BMDC with rIL-4 alone does not confer the potential to induce protective immunity

**Figure 2:**
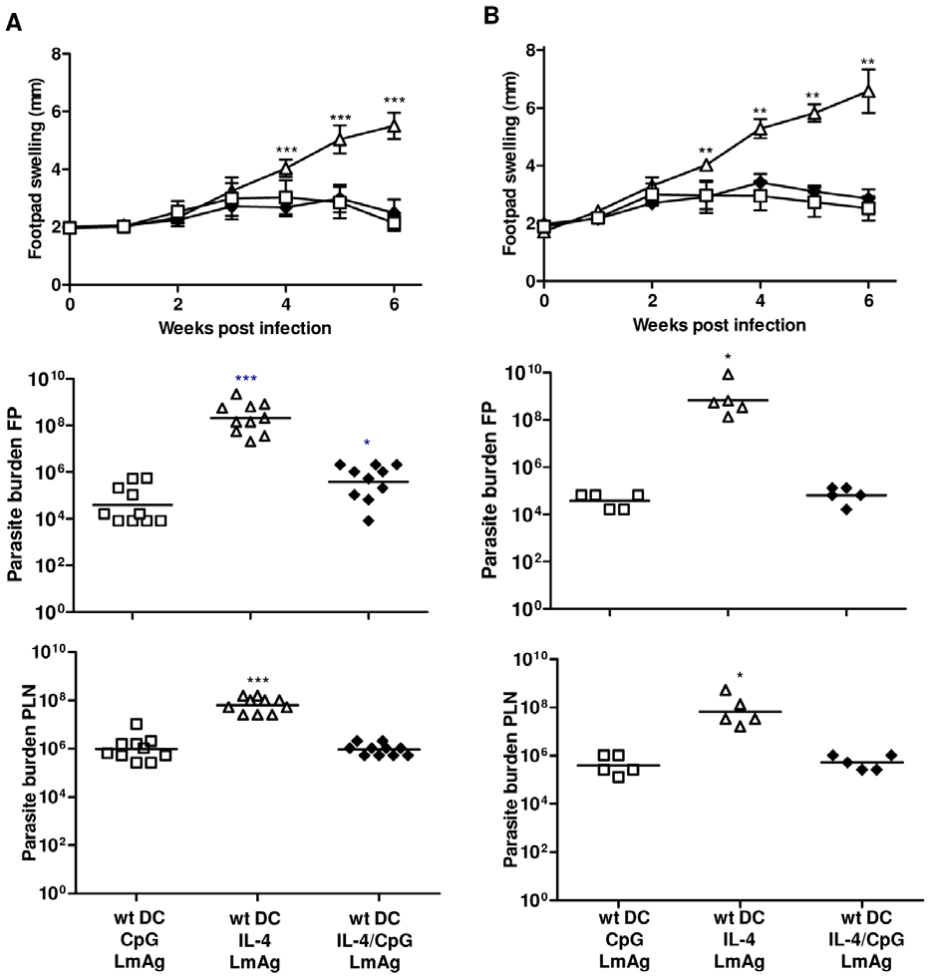
Stimulation of BMDC with rIL-4 prior to LmAg-loading is insufficient to induce protection against leishmaniasis. BALB/c (**A**) or CD11c^cre^IL-4Rα^−/lox^ mice (**B**) were immunized i.v. with rIL-4-stimulated and *L. major* antigen-loaded wt BMDC (wt DC/IL-4/LmAg) (**▵**), a combination of rIL-4 and CpG ODN-activated and *L. major* antigen-loaded wt BMDC (wt DC/IL-4/CpG/LmAg) (⧫) or CpG ODN-activated and *L. major* antigen-loaded wt BMDC (wt DC/CpG/LmAg) (□) one week prior to infection with *L. major*. The footpad swelling was measured weekly. **, p<0.005, ***, p<0.0005 compared to positive control (wt DC/CpG/LmAg) (□). The parasite burden of the infected footpad and the draining popliteal lymph nodes of BALB/c and CD11c^cre^IL-4Rα^−/lox^ mice was determined six weeks after infection. The results of 10 mice (**A**) or 5 mice (**B**) are shown. *, p<0.05, ***, p<0.0005 compared to the respective positive control (wt DC/CpG/LmAg) (□).

To confirm the hypothesis that IL-4Rα signaling is critical for the ability of DC to induce resistance against leishmaniasis and to address the possible combinations of how to activate BMDC used in our vaccination strategy, we used BMDC generated from wt BALB/c mice and stimulated these BMDC with either rIL-4 or CpG ODN alone or a combination of both prior to loading with LmAg. These differently treated BMDC were injected into wt (Figure 2A) or CD11c^cre^IL-4Rα^−/lox^ mice (Figure 2B) one week prior to infection with *L. major*. The course of lesion development was monitored weekly and the parasite burden at the site of infection and the draining lymph node was analyzed. The results show that in the absence of CpG ODN stimulation, rIL-4-treated and LmAg-pulsed BMDC did not have the potential to induce protective immunity with regard to the footpad swelling and the parasite burden in the infected lymph nodes (10^2^-fold increase compared to positive control) and footpad (10^4^-fold increase compared to positive control) in wt (Fig. 2A) or CD11c^cre^IL-4Rα^−/lox^ (Fig. 2B) mice. We did not observe differences in the course of disease in mice immunized with CpG ODN-activated BMDC generated in the presence or absence of rIL-4. Both groups of mice were clinically protected as indicated by controlled footpad swelling and parasite burden in the examined tissues. The results show that additional stimulation of IL-4-responsive BMDC with rIL-4 during vaccine generation seems not to be essential to mediate immunity to *L. major*, but that the boosting effect of additional rIL-4 (see below, Figure 5A) depends on properly activated BMDC.

### IL-4Rα-deficient BMDC are less capable of inducing high levels of activated and mature DC in the draining lymph nodes of infected mice

Host-derived DC migrate to the site of infection, take up and process antigens, which are then loaded onto MHC class I or II molecules. Thus activated, the DC differentiate into mature DC and initiate the immune response while migrating to the local draining lymph nodes, where they cross-talk with other cells of the immune system [22], [23]. For this reason, we analyzed the activation and maturation status of CD11c^+^ cells in the lesion-draining lymph nodes with regard to MHC class II and CD80 expression.

**Figure 3:**
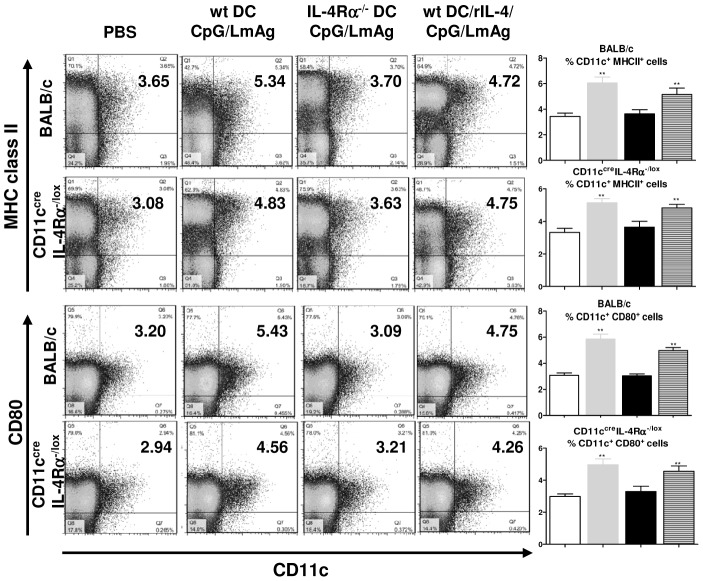
Decreased levels of activated DC in the infected popliteal lymph nodes in mice vaccinated with IL-4Rα^−/−^ BMDC. The lymphocytes of the draining popliteal lymph nodes of 5 BALB/c or 5 CD11c^cre^IL-4Rα^−/lox^ mice, treated as indicated, were collected six weeks post infection, surface-stained for CD11c, MHC class II and CD80 expression to determine the proportion of activated and mature DC in the lymph nodes and analysed using FACSCalibur. The x-axis of the dot blots label CD11c and the y-axis MHC class II or CD80, as indicated. The numbers indicate % of gated cells within the distinct quadrant. The mean ± SD of 5 mice each is shown as bar graphs (white column: PBS-treated group, grey column: wt DC/CpG/LmAg immunized group, black column: IL-4Rα^−/−^ DC/CpG/LmAg immunized group, lined column: wt DC/rIL-4/CpG/LmAg immunized group). **, p<0.005 compared to the respective PBS- treated control group.

Wt or CD11c^cre^IL-4Rα^−/lox^ mice that had been immunized with properly conditioned IL-4Rα-deficient BMDC were less capable of inducing high levels of activated and mature DC in the draining lymph node (Figure 3). Clinically protected mice (immunized with wt DC/CpG/LmAg, grey bars, and wt DC/rIL-4/CpG/LmAg, lined bars) showed significantly higher percentages of CD11c^+^CD80^+^ and CD11c^+^MHCII^+^ cells compared to mice that had been immunized with IL-4Rα^−/−^DC/CpG/LmAg (black bars). These results were indicated in BALB/c mice as early as one week post infection (data not shown).

These results demonstrate that upon immunization with IL-4-responsive BMDC higher percentages of activated and mature recipient DC are observed in lymph nodes of clinically protected mice.

### Vaccination with IL-4Rα-deficient BMDC did not induce a shift towards a protective Th1 response in BALB/c mice

**Figure 4:**
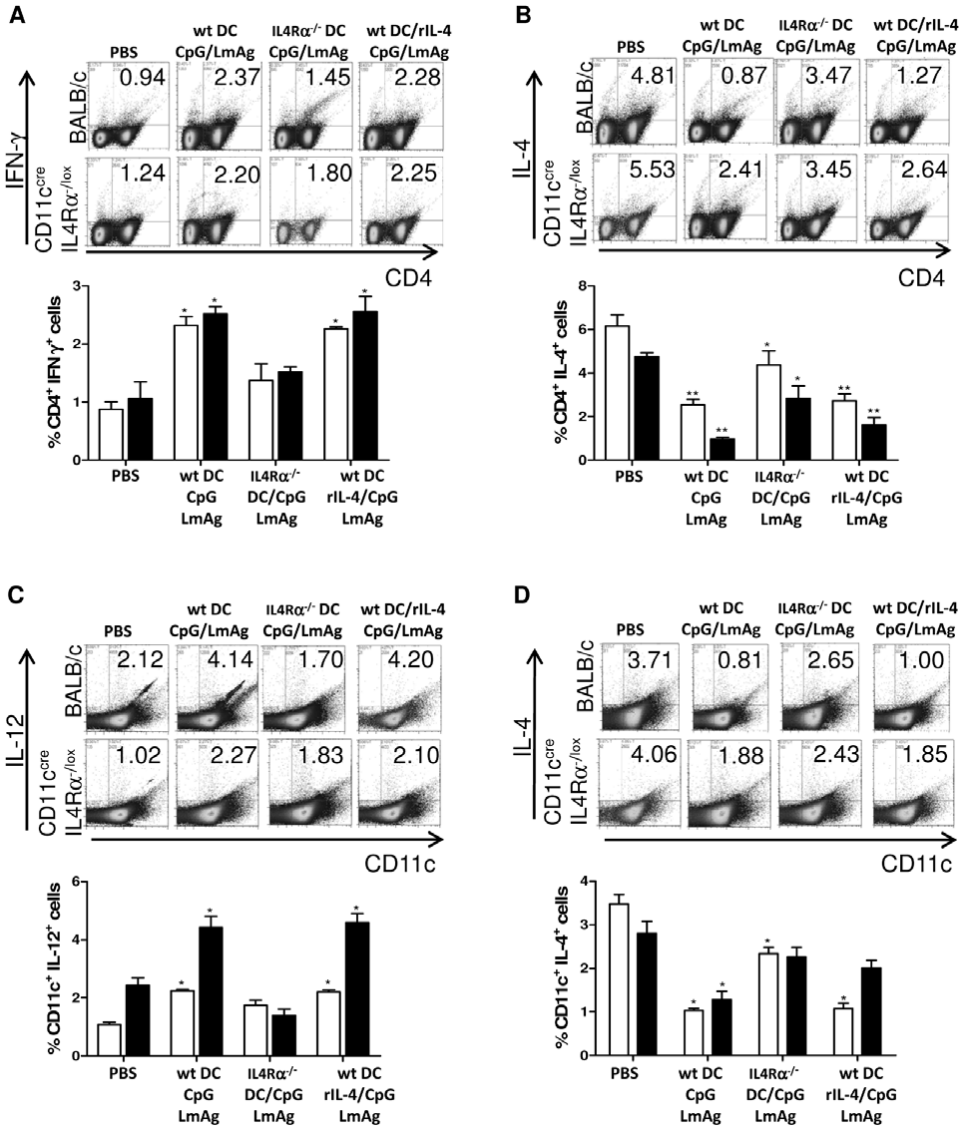
IL-4Rα triggering of vaccine carrier leads to the control of IL-4 production by host lymphocytes. Lymphocytes of the draining popliteal lymph nodes of 5 BALB/c or CD11c^cre^IL-4Rα^−/lox^ mice, treated as indicated, were collected six weeks post infection and activated for 2 hours with PMA-ionomycin before adding monensin for the final 4 hours of culture. The cells were stained for CD4 and IFN-γ (**A**) or IL-4 (**B**) and for CD11c and IL-12 (**C**) or IL-4 (**D**) and analysed using FACSCalibur. The x-axis of the dot blots label CD11c or CD4 and the y-axis IL-4, IL-12 or IFN-γ as indicated. The numbers indicate % of gated cells within the distinct quadrant. The bar graphs show the percentage of gated cytokine-secreting lymphocytes as the mean ± SD of 5 BALB/c (black column) or CD11c^cre^IL-4Rα^−/lox^ mice (white column). *, p<0.05, **, p<0.005, compared to the respective PBS-treated control group.

We analyzed the secretion of IL-4, IL-12 and IFN-γ by CD11c^+^ and CD4^+^ cells in the lymph nodes draining the lesions. Intracellular FACS staining of PMA/ionomycin-stimulated lymphocytes revealed that clinically protected mice have higher levels of Th1 cytokines and low levels of IL-4 in CD11c^+^ and CD4^+^ cells (Figure 4A–D). Both types of vaccinating DC (conditioned wt BMDC in the presence or absence of rIL-4) led to a higher IL-12 secretion by CD11c^+^ cells in the infected lymph nodes compared to unprotected mice (Figure 4C). CD11c^+^ cells of protected mice also displayed lower levels of IL-4 (Figure 4D). In comparison to wt BALB/c mice, CD11c^+^ cells of CD11c^cre^IL-4Rα^−/lox^ mice showed *per se* lower levels of IL-12 and higher levels of IL-4.

CD4^+^ cells secreted lower amounts of IL-4 and higher amounts of IFN-γ in protected mice, compared to unprotected control mice or mice immunized with conditioned IL-4Rα-deficient BMDC (Figure 4A and B). Mice immunized with IL-4Rα-deficient BMDC controlled to a certain level the IL-4 secretion by CD4^+^ cells (Figure 4B), but failed to control IL-4 secretion by CD11c^+^ cells (Figure 4D) and failed to induce high levels of Th1 cytokines by CD11c^+^ (Figure 4C) or CD4^+^ cells (Figure 4A). IL-4 secretion by CD4^+^ cells of CD11c^cre^IL-4Rα^−/lox^ mice is also increased compared to wt BALB/c mice (Figure 4B).

These results demonstrate that IL-4Rα signaling in BMDC used as vaccine carrier enables host DC to secrete high levels of protective IL-12 and, thus, to control IL-4 secretion. This was already indicated at 3 weeks post infection (data not shown).

### IL-4Rα signaling in BMDC used as vaccine carrier is important for the *L. major*-induced release of Th1 cytokines

Protection against leishmaniasis is associated with a Th1 immune response characterized by high levels of IL-12 and low levels of IL-4. To analyze the potential of BMDC-based vaccines to mediate a *L. major*-stimulated Th1 response, total lymphocytes of all groups were collected six weeks post infection and stimulated for 72 hours with LmAg. Subsequently, the cytokine levels of IL-4, IL-12 and IFN-γ were measured by ELISA.

**Figure 5:**
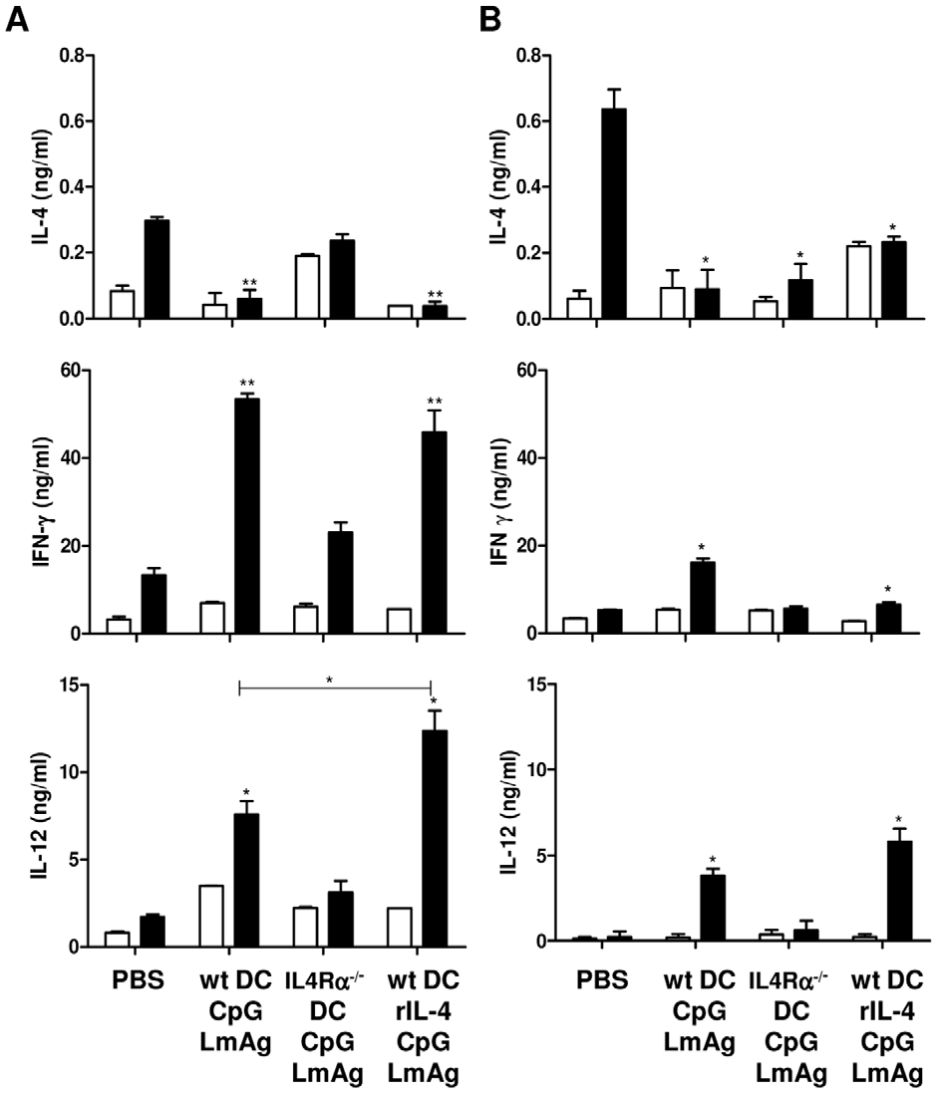
IL-4Rα triggering of BMDC increases *L. major*-stimulated IL-12 secretion *in vivo*. BALB/c (**A**) or CD11c^cre^IL-4Rα^−/lox^ mice (**B**) were immunized with 5×10^5^ BMDC prepared as indicated and infected one week later with 2×10^5^ *L. major* promastigotes. Total lymphocytes of the draining popliteal lymph nodes were collected six weeks post infection and incubated for 72 hours in the absence (white bars) or presence (black bars) of LmAg. The levels of IL-4, IFN-γ and IL-12 were measured by ELISA in the collected supernatants. The results of 5 mice are shown. *, p<0.05, **, p<0.005 compared to the respective PBS-treated control group.

LmAg stimulation of lymph node cells from protected BALB/c mice caused the secretion of high levels of IL-12 and IFN-γ and low levels of IL-4, whereas mice immunized with IL-4Rα^−/−^ DC showed a reversed cytokine pattern (Figure 5A). These results were already indicated at one week post infection (data not shown). Interestingly, the additional stimulation of properly activated IL-4-responsive BMDC with rIL-4 resulted in elevated levels of IL-12 upon *L. major* infection *in vivo* in BALB/c mice.

Low levels of IL-4 were observed in CD11c^cre^IL-4Rα^−/lox^ mice independent of the presence or absence of IL-4Rα on DC used for immunization, as in contrast to BALB/c mice, also conditioned IL-4Rα^−/−^ BMDC were able to inhibit the release of IL-4 upon *L. major* stimulation (Figure 5B). In line with the results obtained with BALB/c mice, increased levels of IFN-γ were observed in mice immunized with properly conditioned wt BMDC, resulting in protection against *L. major*. Immunization with conditioned IL-4Rα^−/−^ BMDC was unable to induce the production of IL-12 and IFN-γ, even though the *L. major*-stimulated IL-4 secretion was controlled.

Elevated levels of IL-12 were only observed in wt BALB/c mice upon immunization with properly activated and additionally rIL-4-stimulated BMDC, hence indicating the role of IL-4-responding host DC in the induction of IL-12 release upon *L. major* infection. As already shown in Figure 4, wt BALB/c mice secrete higher levels of Th1 cytokines, whereas CD11c^cre^IL-4Rα^−/lox^ mice secrete higher levels of IL-4. A complete IL-4Rα-deficient set-up (vaccine and host) showed that IL-4Rα-mediated instruction of DC is important to enhance protection against leishmaniasis, as IL-4Rα-deficient DC were not capable of mediating resistance in CD11c^cre^IL-4Rα^−/lox^ mice.

## Discussion

In the present study, we investigated the importance of IL-4Rα triggering during DC-mediated vaccination against the protozoan parasite *L. major*. The results show that complete protection against otherwise lethal leishmaniasis required immunization of BALB/c mice with IL-4-responsive BMDC, while IL-4Rα^−/−^ BMDC failed to induce the restriction of lesion development. Even though the footpad swelling was restricted during the first three weeks of infection, a progressive course of disease with development of severe and necrotic lesions was observed at later stages of infection. *In vitro* studies showed that IL-4Rα-deficient BMDC secrete lower amounts of IL-12 and higher amounts of IL-10 upon stimulation with CpG ODN and LmAg compared to wt BMDC, which is most probably the reason for the failure of immunization with IL-4Rα-deficient BMDC, as no differences were observed regarding the activation status of wt or IL-4Rα-deficient BMDC (data not shown). Importantly, the levels of *Leishmania*-stimulated IL-12, the most potent inducer of immunity to *L. major* [24], [25], were significantly increased in the lymph nodes of wt BALB/c mice immunized with properly conditioned wt BMDC that had been additionally activated with rIL-4. Elevated levels of IL-12 upon stimulation with LmAg were only observed in IL-4 responder recipients (wt BALB/c), but not in CD11c^cre^IL-4Rα^−/lox^ mice, demonstrating that IL-4 instruction of host DC is required to induce elevated levels of IL-12 during *L. major* infection. BMDC activated with rIL-4 alone were not able to mediate protection against *L. major*, but induced elevated *Leishmania*-stimulated IL-4 levels *in vivo* (data not shown), skewing CD4^+^ T cells towards a Th2 cell phenotype and promoting susceptibility in BALB/c mice.

At the site of infection, neutrophils instruct DC recruitment and activation, leading to Th1 cell activation and immunity to microbial infection [26]. Our results extend these findings by showing that upon immunization with IL-4-responsive DC, higher percentages of activated and mature recipient DC are observed in the lymph nodes draining the site of infection. In contrast, a less pronounced increase of mature DC is found upon immunization with IL-4 non-responder DC.

The Th1/Th2 paradigm of experimental leishmaniasis is associated with IL-12- and IFN-γ-mediated resistance or IL-4-mediated susceptibility to *L. major* infection [2]. It is commonly accepted that IL-4 is the hallmark cytokine mediating the differentiation of naïve Th0 cells into the Th2 phenotype. However, the point that an IL-4-mediated Th2 response renders mice necessarily susceptible has never been proven for visceral leishmaniasis [27]. Furthermore, several data revealed a Th1-promoting effect of IL-4 which is capable to prime for bioactive IL-12. For example, treating human peripheral blood mononuclear cells (PBMC) with IL-4 enhanced their IL-12 response to lipopolysaccharide or *Staphylococcus aureus* [28], IL-12 production by human monocytes during interaction with T cells was increased upon IL-4 stimulation [29] and IL-4 provided a negative feedback causing murine as well as human DC to produce IL-12 [30]. IL-4 was furthermore reported to be required for the induction of protective Th1 cell responses to fungal infections, such as *Candida albicans* [31].

A protective role of IL-4 has also been shown for *L. major* infection in susceptible BALB/c mice [14]. It is important to note that the resistance-promoting role of IL-4 was only achieved when IL-4 was strictly present during the initial activation of DC upon infection. The presence of IL-4 during T cell priming resulted in the development of Th2 cells, which even rendered resistant TCR V_β_4-deficient BALB/c mice susceptible to leishmaniasis. IL-4 acting on DC induced the generation of a protective Th1 immune response against leishmaniasis in BALB/c mice [14].

Furthermore, it has been demonstrated that endogenous IL-4 is necessary for effective drug therapy with sodium stibogluconate against visceral leishmaniasis in BALB/c mice, as IL-4-deficient mice responded poorly to this treatment and showed increased parasite burdens in infected tissues [32]. Another example for IL-4-promoted healing has been documented in BALB/c mice vaccinated with a liposomal formulation against *L. donovani*, where an initially vaccine-induced mixed Th1/Th2 response, characterized by high levels of IFN-γ and IL-4, instructed an efficient Th1-mediated resistance [33]. Our data are consistent with these studies, showing that IL-4Rα signaling is important to enable DC to induce a protective immune response in the recipient mice, hallmarked by high levels of *L. major*-induced IL-12 production in the lymph nodes of infected IL-4 responder mice.

Elevated IL-4 levels during the late phase of *L. major* infection in resistant C57BL/6 mice were associated with the maintenance of an existing protection [34], whereas susceptible BALB/c mice showed elevated IL-4 levels only during the early phase of infection [35]. These findings suggested a role of IL-4 in sustaining protection during the chronic phase of leishmaniasis.

The results of the present study indicate a direct link between IL-4Rα triggering of BMDC used for immunization and the induction of elevated levels of IL-12 upon *L. major* infection in BALB/c mice, which mediated complete protection against otherwise lethal leishmaniasis. Resistance in leishmaniasis has been reported to depend on DC-derived IL-12 [11], the inhibition of *Leishmania*-specific IL-4-secretion by Vβ4Vα8 CD4^+^ T cells and the induction of a Th1-dominated immune response *in vivo* [36]. Another aspect to give consideration to is that immunizing CD11c^cre^IL-4Rα^−/lox^ mice with IL-4Rα^−/−^ DC resulted in progressive leishmaniasis, showing the importance of IL-4Rα signaling not only in the immunizing DC but also in the host DC. This complete IL-4Rα-deficient DC set-up caused uncontrolled parasite dissemination into the draining lymph node, indicating that the inhibition of IL-4Rα signaling in host DC is detrimental and leads to increased dissemination of parasites into organs. In general, CD11c^cre^IL-4Rα^−/lox^ mice secrete elevated levels of IL-4 and decreased Th1 cytokines compared to wt BALB/c mice and the effect of increased IL-12 secretion upon immunization with additionally rIL-4-stimulated wt BMDC was not observed in these mice, showing the important role of IL-4Rα signaling in host DC for IL-12 production during *L. major* infection. Immunized CD11c^cre^IL-4Rα^−/lox^ mice showed controlled IL-4 levels independent of the type of vaccine, while IL-4Rα-deficient BMDC failed to induce a protective Th1 cytokine profile, resulting in a nonprotective Th2 immune response. These results showed that IL-4Rα signaling in the DC vaccine carrier is more critical than the IL-4 responsiveness of host DC.

The present study enhances our understanding of the role of IL-4Rα signaling in DC during cell-mediated vaccination against an intracellular pathogen by showing that triggering this receptor is essential to confer protection. Vaccination strategies against Th2-related diseases, such as allergies or parasitic infections, should not only concentrate on inhibiting anti-inflammatory Th2 responses by inducing a strong Th1 phenotype, but need to consider the proinflammatory effect of IL-4 as adjuvant on the vaccine efficiency. An important aspect to be considered is that IL-4 as well as IL-13 can signal through the common IL-4Rα chain. *In vitro* results showed that wt BMDC stimulated with CpG ODN and IL-4, but not IL-13, induced the secretion of elevated IL-12 levels compared to CpG ODN stimulated wt BMDC, and that additional stimulation with IL-4 or IL-13 failed to induce elevated levels of IL-12 secretion by IL-4Rα-deficient BMDC (data not shown). These *in vitro* results strongly suggest that the elevated secretion of DC-derived IL-12 is induced by IL-4 instruction of DC and not by IL-13 instruction (see also Hurdayal et al., manuscript in preparation). The results of the present study underline the importance of IL-4 signaling during vaccine design, as IL-4Rα signaling in the DC vaccine carrier is more important than IL-4Rα signaling in the host DC. Our results document the crucial role of IL-4Rα signaling in DC-based vaccination against leishmaniasis by promoting a protective Th1 immune response.
